# Association of glycemic status and segmental left ventricular wall thickness in subjects without prior cardiovascular disease: a cross-sectional study

**DOI:** 10.1186/s12872-018-0900-7

**Published:** 2018-08-09

**Authors:** Susanne Rospleszcz, Anina Schafnitzel, Wolfgang Koenig, Roberto Lorbeer, Sigrid Auweter, Cornelia Huth, Wolfgang Rathmann, Margit Heier, Birgit Linkohr, Christa Meisinger, Holger Hetterich, Fabian Bamberg, Annette Peters

**Affiliations:** 1 Institute of Epidemiology, Helmholtz Zentrum München, German Research Center for Environmental Health, Ingolstaedter Landstrasse 1, 85764 Neuherberg, Germany; 2 Department of Radiology, Ludwig-Maximilians-University Hospital, Munich, Germany; 3 Department of Internal Medicine II – Cardiology, University of Ulm Medical Center, Ulm, Germany; 4 Deutsches Herzzentrum München, Technische Universität München, Munich, Germany; 5 German Centre for Cardiovascular Research (DZHK e.V.), Munich, Germany; 6 German Center for Diabetes Research (DZD), München-Neuherberg, Germany; 7 Institute for Biometrics and Epidemiology, German Diabetes Center, Duesseldorf, Germany; 8 KORA Myocardial Infarction Registry, Central Hospital of Augsburg, Augsburg, Germany; 9 Department of Diagnostic and Interventional Radiology, University of Tuebingen, Tuebingen, Germany

**Keywords:** Cardiac magnetic resonance imaging, Prediabetes, Diabetes, Left ventricular wall thickness, 16-segment model

## Abstract

**Background:**

Left ventricular (LV) hypertrophy and changes in LV geometry are associated with increased cardiovascular mortality. Subjects with type 2 diabetes have an increased risk of such alterations in cardiac morphology. We sought to assess the association of glycemic status and LV wall thickness measured by cardiac magnetic resonance (CMR), and potential interactions of hypertension and diabetes.

**Methods:**

CMR was performed on 359 participants from a cross-sectional study nested in a population-based cohort (KORA FF4) free of overt cardiovascular disease. Participants were classified according to their glycemic status as either control (normal glucose metabolism), prediabetes or type 2 diabetes. Segmentation of the left ventricle was defined according to the American Heart Association (AHA) 16-segment model. Measurements of wall thickness were obtained at end-diastole and analyzed by linear regression models adjusted for traditional cardiovascular risk factors.

**Results:**

LV wall thickness gradually increased from normoglycemic controls to subjects with prediabetes and subjects with diabetes (8.8 ± 1.4 vs 9.9 ± 1.4 vs 10.5 ± 1.6 mm, respectively). The association was independent of hypertension and traditional cardiovascular risk factors (β-coefficient: 0.44 mm for prediabetes and 0.70 mm for diabetes, *p*-values compared to controls: *p* = 0.007 and *p* = 0.004, respectively). Whereas the association of glycemic status was strongest for the mid-cavity segments, the association of hypertension was strongest for the basal segments.

**Conclusion:**

Abnormal glucose metabolism, including pre-diabetes, is associated with increased LV wall thickness independent of hypertension.

**Electronic supplementary material:**

The online version of this article (10.1186/s12872-018-0900-7) contains supplementary material, which is available to authorized users.

## Background

Abnormal cardiac morphology, such as left ventricular (LV) hypertrophy and altered geometry, represents a risk factor for increased cardiovascular mortality and morbidity [1, 2].

Increased LV wall thickness is considered as an adaptive response to augmented wall stress caused by pressure overload. Short-term increase in wall thickness can therefore be regarded as a beneficial adaptation in order to maintain oxygen demand and contractile function of the heart [3]. However, a persistent increase in wall thickness leads to impaired myocardial relaxation and subsequently to decreased diastolic function, [4] which is associated with diastolic heart failure and other adverse cardiovascular outcomes [5]. The exact pathophysiological pathways of the transition from compensatory response to a detrimental chronic condition are not yet fully understood.

Chronic hypertension and the resulting increased hemodynamic load are a major risk factor for cardiac remodeling. However, metabolic factors, including diabetes status, play an important role [6–9]. Multiple studies have used echocardiography to analyze the potential impact of glycemic status on measures of LV mass and geometric patterns. Although most studies found higher values of LV mass in people with abnormal glucose metabolism, these associations were often attenuated by the presence of other traditional cardiovascular risk factors, especially elevated blood pressure [10–15]. Moreover, the prognostic utility of LV mass for CVD events in people with diabetes also depends on other metabolic factors [16–18].

These findings raise the question whether these measurements of LV mass and geometric patterns are detailed enough to describe the complex interplay between glycemic status and blood pressure on cardiac structure. Cardiac magnetic resonance imaging (CMR) has now become the gold standard for the measurement of myocardial mass and volumes [19, 20] and delivers a more detailed characterization of cardiac morphology than echocardiography, thereby allowing more precise insights into the mechanisms of LV remodeling.

In our initial analyses, using CMR to derive measures of LV geometry and function, we had observed an increased myocardial mass in subjects with abnormal glucose metabolism, but the difference disappeared after adjustment for major cardiovascular risk factors [21]. We therefore aim to elucidate the impact of glycemic status on regional LV remodeling and further analyze its potential interaction with blood pressure.

## Methods

### Study sample

The study sample is a subsample of the second follow-up of the population-based KORA (“Cooperative Health Research in the Region of Augsburg”) S4 cohort (KORA FF4). The major focus of the substudy is the analysis of subclinical cardiovascular disease by whole-body magnetic resonance imaging (MRI).

Recruitment and eligibility criteria for the KORA studies have been described elsewhere [22]. The KORA FF4 study was conducted between 2013 and 2014 and included 2279 of the originally recruited 4261 KORA S4 participants. Of these, 400 subjects participated in the MRI substudy who were eligible and willing to undergo whole-body MRI. The detailed participant flow and exclusion criteria have been described previously [21].

Additionally, we excluded 31 subjects with incomplete measurements of any AHA segment due to low image quality and subsequently excluded 10 subjects with visible Late Gadolinium Enhancement.

### Covariate assessment

Glycemic status was defined as known diabetes, either self-reported or defined by current use of glucose-lowering medication, and in participants without known diabetes, it was determined by a standard 2-h oral glucose tolerance test (OGTT). According to the 1999 WHO criteria [23], subjects with fasting serum glucose levels ≥7.0 mmol/l or OGTT 2-h serum glucose levels ≥11.1 mmol/l were also classified as having diabetes. Isolated impaired fasting glucose (iIFG) was defined as fasting glucose ≥6.1 but < 6.9 mmol/l and 2-h glucose < 7.8 mmol/l. Isolated impaired glucose tolerance (iIGT) was defined as fasting glucose < 6.1 mmol/l and 2-h glucose ≥7.8 but < 11.1 mmol/l. Subjects with iIFG, iIGT or with both conditions were classified as having prediabetes. Subjects with fasting glucose < 6.1 mmol/l and 2-h glucose < 7.8 mmol/l were considered controls.

Hypertension was defined as current antihypertensive treatment and/or systolic/diastolic blood pressure above 140/90 mmHg. Prehypertension was defined as systolic/diastolic blood pressure above 120/80 mmHg. Subjects were classified as smokers if they reported current regular or sporadic cigarette smoking. Cholesterol, serum glucose, serum insulin and Hba1c were determined by standard methods as described in Additional file 1: Text S1.

### CMR outcome assessment

Magnetic resonance imaging was performed at a 3 Tesla Magnetom Skyra (Siemens AG, Healthcare Sector, Erlangen Germany) using a 18 channel body coil in combination with the table-mounted spine matrix coil. The whole-body MRI protocol comprised several sequences as described previously [21].

Imaging of cardiac function and morphology was performed using cine steady-state free precession (cine-SSFP) sequences in the short axis with a stack of 10 layer and 25 phases per cardiac cycle as well as in a 4-chamber view (echo time 1.46 ms, repetition time 29.97 ms, in-plane voxel size 1.5 × 1.5 mm, flip angle 62–63°, field-of-view 297 × 360 mm, matrix size 240 × 160 mm, slice thickness 8 mm).

The cine-SSFP sequences were then analyzed semi-automatically using cvi42 software (Circle Cardiovascular Imaging, Calgary, Canada) by two readers unaware of the subject’s glycemic status and all other clinical covariates. In the 4-chamber view, apex and base of the LV were first manually selected, followed by automatic border detection of the LV endocardial and epicardial border in the short axis. Borders were corrected manually, if necessary. The basal slice was selected when at least 50 % of the LV cavity was surrounded by myocardium at end-diastole. Papillary muscles and trabeculations were excluded from the myocardial area and included in the blood pool. To assess intraobserver agreement, measurements of 25 randomly chosen subjects were repeated by the first reader. Interobserver agreement was assessed on 52 subjects who were measured by the first and the second reader. Intra- and Interobserver agreement were calculated by the Intraclass Correlation Coefficient (ICC).

Mean wall thickness was measured at the end of diastole for each segment according to the American Heart Association (AHA) 16-segment model [24]. Measurements of the single segments are visualized in a polar plot according to glycemic status. For further statistical analysis, segments are grouped according to level (basal: AHA segments 1–6; mid-cavity: AHA segments 7–12; apical: AHA segments 12–16) and region (lateral: AHA segments 5, 6, 11, 12 and 16; septal: AHA segments 2, 3, 8 and 9; anterior: AHA segments 1, 7 and 13; inferior: AHA segments 4, 10 and 15) [24].

### Statistical analysis

Main demographic and cardiovascular characteristics of the participants are reported as arithmetic means with standard deviations for continuous variables and counts and percentages for categorical variables. Differences in wall thickness between the glycemic groups were assessed by one-way ANOVA. A linear regression model including glycemic status, age, sex, Body Mass Index (BMI), systolic blood pressure, total cholesterol, use of antihypertensive medication and smoking status was calculated to determine the association of glycemic status to the respective wall thickness variable. The same model without adjustment for systolic blood pressure was calculated for hypertension. Additionally, linear regression models with multiplicative terms between glycemic status and systolic blood pressure were computed to discover any interaction effects.

As the MRI sample is a non-representative subsample of a population based cohort, we used appropriate sampling weights to render the sample more representative of the full eligible underlying cohort. Weighting was based on glycemic status, age and sex. Details of the weighting procedure are presented in Additional file 1: Text S2.

*P*-values < 0.05 were considered to denote statistical significance. All analyses were done with R version 3.2.1 (R Core Team, Vienna, Austria).

The KORA FF4 study was approved by the ethics committee of the Bavarian Chamber of Physicians, Munich; the MRI substudy by the institutional review board of the Ludwig-Maximilians-University Munich. The investigations were carried out in accordance with the Declaration of Helsinki, including written informed consent of all participants.

## Results

### Study population

The sample of 359 subjects comprised 223 normoglycemic controls (62%), 92 subjects with prediabetes (26%) and 44 subjects with diabetes (12%) as presented in Table 1. Of those, 15 diabetes cases were diagnosed based on the results of OGTT. In subjects with established diabetes the median duration of diabetes was 7.0 years.

**Table 1:** Demographic characteristics of study participants

	All	Control	Prediabetes	Type 2 diabetes
	*N* = 359	*N* = 223	*N* = 92	*N* = 44
Age (years)	56.1 ± 9.1	54.3 ± 8.9	58.1 ± 8.8	61.4 ± 8.3
Male gender	205 (57.1%)	115 (51.6%)	58 (63.0%)	32 (72.7%)
BMI (kg/m^2^)	27.9 ± 4.8	26.5 ± 4.2	30.3 ± 4.6	30.2 ± 5.1
Systolic blood pressure (mmHg)	120.3 ± 16.9	116.5 ± 15.2	124.7 ± 15.5	130.1 ± 21.2
Diastolic blood pressure (mmHg)	75.3 ± 10.1	73.7 ± 9.2	78.0 ± 9.7	78.0 ± 13.2
Prehypertension	94 (26.2%)	61 (27.4%)	26 (28.3%)	7 (15.9%)
Hypertension	117 (32.6%)	45 (20.2%)	41 (44.6%)	31 (70.5%)
Fasting glucose (mmol/L)	5.7 ± 1.3	5.2 ± 0.4	5.9 ± 0.6	8.0 ± 2.3^a^
Fasting insulin (pmol/L)	65.4 ± 41.1	51.6 ± 26.7	86.9 ± 44.6	91.7 ± 60.0^b^
HbA1c (%)	5.6 ± 0.7	5.3 ± 0.3	5.6 ± 0.3	6.7 ± 1.4
Total cholesterol (mmol/L)	5.6 ± 1.0	5.6 ± 0.9	5.8 ± 0.8	5.5 ± 1.2
HDL cholesterol (mmol/L)	1.6 ± 0.5	1.7 ± 0.5	1.5 ± 0.4	1.4 ± 0.4
LDL cholesterol (mmol/L)	3.6 ± 0.9	3.6 ± 0.8	3.7 ± 0.8	3.5 ± 1.1
Triglycerides (mmol/L)	1.5 ± 1.0	1.2 ± 0.7	1.7 ± 1.0	2.3 ± 1.4
Smoking				
Never-smoker	130 (36.2%)	88 (39.5%)	28 (30.4%)	14 (31.8%)
Ex-smoker	156 (43.5%)	87 (39.0%)	46 (50.0%)	23 (52.3%)
Smoker	73 (20.3%)	48 (21.5%)	18 (19.6%)	7 (15.9%)
Antihypertensive medication	85 (23.7%)	35 (15.7%)	28 (30.4%)	22 (50.0%)

Values are arithmetic means ± standard deviations for continuous variables and number of subjects (percentage of respective group) for categorical outcomes

^a^Based on *N* = 43 subjects with type 2 diabetes

^b^Based on *N* = 42 subjects with type 2 diabetes

### Intra- and Interobserver agreement

The ICCs for intraobserver and interobserver agreement were 0.93 and 0.94 for mean wall thickness, respectively. ICCs for single segments are presented in Additional file 1: Figure S1.

### Comparison of wall thickness according to glycemic status

**Fig. 1:**
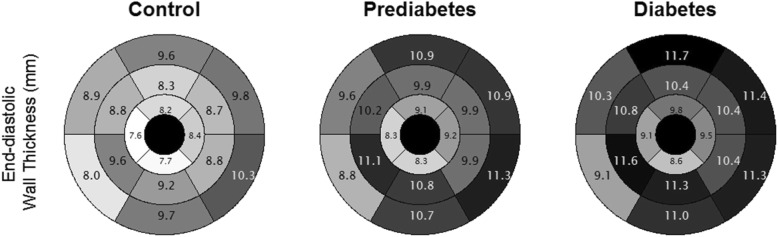
Left ventricular wall thickness (mm) of 16 AHA segments for control, prediabetes and diabetes group

Mean wall thickness of all AHA segments in the whole sample was 9.1 mm (± standard deviation: 1.5 mm). The polar plots in Fig. 1 display the wall thickness of the individual AHA segments for the three glycemic groups.

We found a gradual increase in wall thickness from controls through prediabetes to diabetes for all classes of segments grouped by level and region. The differences between the glycemic groups were statistically significant in univariate analysis for all analyzed segment classes (Table 2).

**Table 2:** Mean wall thickness grouped by level and region and myocardial mass

	All	Control	Prediabetes	*P*-value	Type 2 diabetes	*P*-value
	*N* = 359	*N* = 223	*N* = 92		*N* = 44	
Wall thickness (mm): arithmetic mean of						
All segments	9.1 ± 1.5	8.8 ± 1.4	9.9 ± 1.4	**< 0.001**	10.5 ± 1.6	**< 0.001**
Basal segments	9.6 ± 1.6	9.4 ± 1.6	10.4 ± 1.6	**< 0.001**	10.8 ± 1.7	**< 0.001**
Mid segments	9.2 ± 1.8	8.9 ± 1.6	10.3 ± 1.8	**< 0.001**	10.9 ± 2.1	**< 0.001**
Apical segments	8.2 ± 1.5	8.0 ± 1.4	8.7 ± 1.5	**< 0.001**	9.3 ± 1.6	**< 0.001**
Lateral segments	9.8 ± 1.6	9.3 ± 1.5	10.3 ± 1.4	**< 0.001**	10.8 ± 1.8	**< 0.001**
Septal segments	9.1 ± 1.6	8.7 ± 1.5	9.7 ± 1.4	**< 0.001**	10.3 ± 1.6	**< 0.001**
Anterior segments	9.4 ± 1.9	8.8 ± 1.7	10.0 ± 1.7	**< 0.001**	10.8 ± 2.2	**< 0.001**
Inferior segments	9.4 ± 1.5	9.0 ± 1.5	9.9 ± 1.4	**< 0.001**	10.4 ± 1.4	**< 0.001**
Myocardial mass (g/m^2^)	70.0 ± 13.9	69.0 ± 13.6	72.3 ± 13.0	0.1	75.9 ± 16.0	**0.019**

*P*-values are obtained from one-way ANOVA and Bonferroni corrected for the repeated comparisons to the control group

### Association of glycemic status and wall thickness independent of confounding factors

After adjustment for additional covariates as detailed above, prediabetes and diabetes were independently associated to increased wall thickness (prediabetes: β: 0.44 mm, 95%-CI: [0.12 mm, 0.75 mm], diabetes: β: 0.70 mm, 95%-CI: [0.23 mm, 1.17 mm]). Associations held true for most segment classes according to level and region as presented in Table 3. The strongest associations were found for the mid-cavity segments and the anterior segments.

**Table 3:** Association of glycemic status and wall thickness

	Prediabetes			Diabetes		
	Estimate	95%-CI	*P*-value	Estimate	95%-CI	*P*-value
Wall thickness (mm): arithmetic mean of						
All segments	0.44	[0.12, 0.75]	**0.007**	0.70	[0.23, 1.17]	**0.004**
Basal segments	0.33	[−0.05, 0.70]	0.087	0.51	[0.02, 0.99]	**0.040**
Mid segments	0.61	[0.20, 1.02]	**0.004**	0.86	[0.28, 1.45]	**0.004**
Apical segments	0.34	[−0.04, 0.73]	0.080	0.74	[0.23, 1.24]	**0.005**
Lateral segments	0.46	[0.09, 0.83]	**0.014**	0.65	[0.14, 1.16]	**0.013**
Septal segments	0.35	[0.05, 0.65]	**0.023**	0.64	[0.18, 1.10]	**0.006**
Anterior segments	0.52	[0.10, 0.93]	**0.015**	0.98	[0.38, 1.59]	**0.002**
Inferior segments	0.45	[0.11, 0.79]	**0.009**	0.58	[0.11, 1.04]	**0.016**
Myocardial mass (g/m^2^)	−0.11	[−3.51, 3.28]	0.948	0.56	[−4.94, 6.07]	0.841

Estimates from linear regression models adjusted for age, sex, BMI, systolic blood pressure, total cholesterol, use of antihypertensive medication and smoking status

### Effects of weighting

Additional file 1: Table S1 shows the characteristics of the underlying eligible cohort that was used for the calculation of sampling weights.

Comparing the MRI sample to the whole cohort, there was an overrepresentation of subjects with prediabetes (26% in the sample vs 12% in the cohort) and subjects with diabetes (12% in the sample vs 10% in the cohort). Additionally, the proportion of males was higher in the MRI sample compared to the underlying cohort, whereas mean age was similar.

Results of the unweighted analysis are presented in Additional file 1: Table S2. Associations that were statistically significant in the weighted analysis were also significant in the unweighted analysis. The size of the estimates was comparable; however as sampling weights introduce additional variation in the data, confidence intervals for the weighted analysis were wider than for the unweighted analysis. Model diagnostics such as residual distribution were similar between the weighted and unweighted analysis. Taken together, the evidence suggests that the analytical model was correctly specified [25] and although the weighted estimates are conceptually more precise, as they relate to the underlying cohort, the actual differences between weighted and unweighted estimates were small.

### Association of specific prediabetes subgroups and wall thickness

We further differentiated prediabetes status into subjects with iIFG (*N* = 35, 9.7% of total sample and 38.1% of subjects with prediabetes), iIGT (*N* = 41, 11.4% of total sample and 44.6% of subjects with prediabetes) and both IFG + IGT (*N* = 16, 4.5% of total sample and 17.4% of subjects with prediabetes). Though there were differences in mean wall thickness according to segment classes between the prediabetes subgroups there was no gradual increase from iIFG to iIGT and IFG + IGT (See Additional file 1: Figure S2 and Table S3).

### Association of hypertension and wall thickness independent of confounding factors

Prehypertension and hypertension were significantly associated with increased wall thickness (prehypertension: β: 0.48 mm, 95%-CI: [0.17 mm, 0.79 mm], hypertension: β: 0.67 mm, 95%-CI: [0.31 mm, 1.02 mm]) after adjustment for additional covariates. The strongest associations were seen for the basal segments and the septal segments as presented in Table 4. Notably, there was also a significant association of hypertension to myocardial mass.

**Table 4:** Association of hypertension and wall thickness

	Prehypertension			Hypertension		
	Estimate	95%-CI	*P*-value	Estimate	95%-CI	*P*-value
Wall thickness (mm): arithmetic mean of						
All segments	0.48	[0.17, 0.79]	**0.003**	0.67	[0.31, 1.02]	**< 0.001**
Basal segments	0.63	[0.28, 0.98]	**< 0.001**	0.83	[0.42, 1.24]	**< 0.001**
Mid segments	0.35	[−0.01, 0.71]	0.057	0.70	[0.26, 1.14]	**0.002**
Apical segments	0.44	[0.02, 0.86]	**0.043**	0.38	[−0.01, 0.77]	0.060
Lateral segments	0.37	[0.03, 0.71]	**0.036**	0.52	[0.12, 0.92]	**0.012**
Septal segments	0.59	[0.29, 0.89]	**< 0.001**	0.84	[0.48, 1.19]	**< 0.001**
Anterior segments	0.44	[0.02, 0.86]	**0.039**	0.77	[0.34, 1.19]	**< 0.001**
Inferior segments	0.51	[0.14, 0.88]	**0.007**	0.54	[0.16, 0.92]	**0.006**
Myocardial mass (g/m^2^)	3.18	[−0.02, 6.39]	0.053	6.16	[2.19, 10.12]	**0.003**

Estimates from linear regression models adjusted for age, sex, BMI, glycemic status, total cholesterol, use of antihypertensive medication and smoking status

### Interaction of glycemic status and blood pressure

**Fig. 2:**
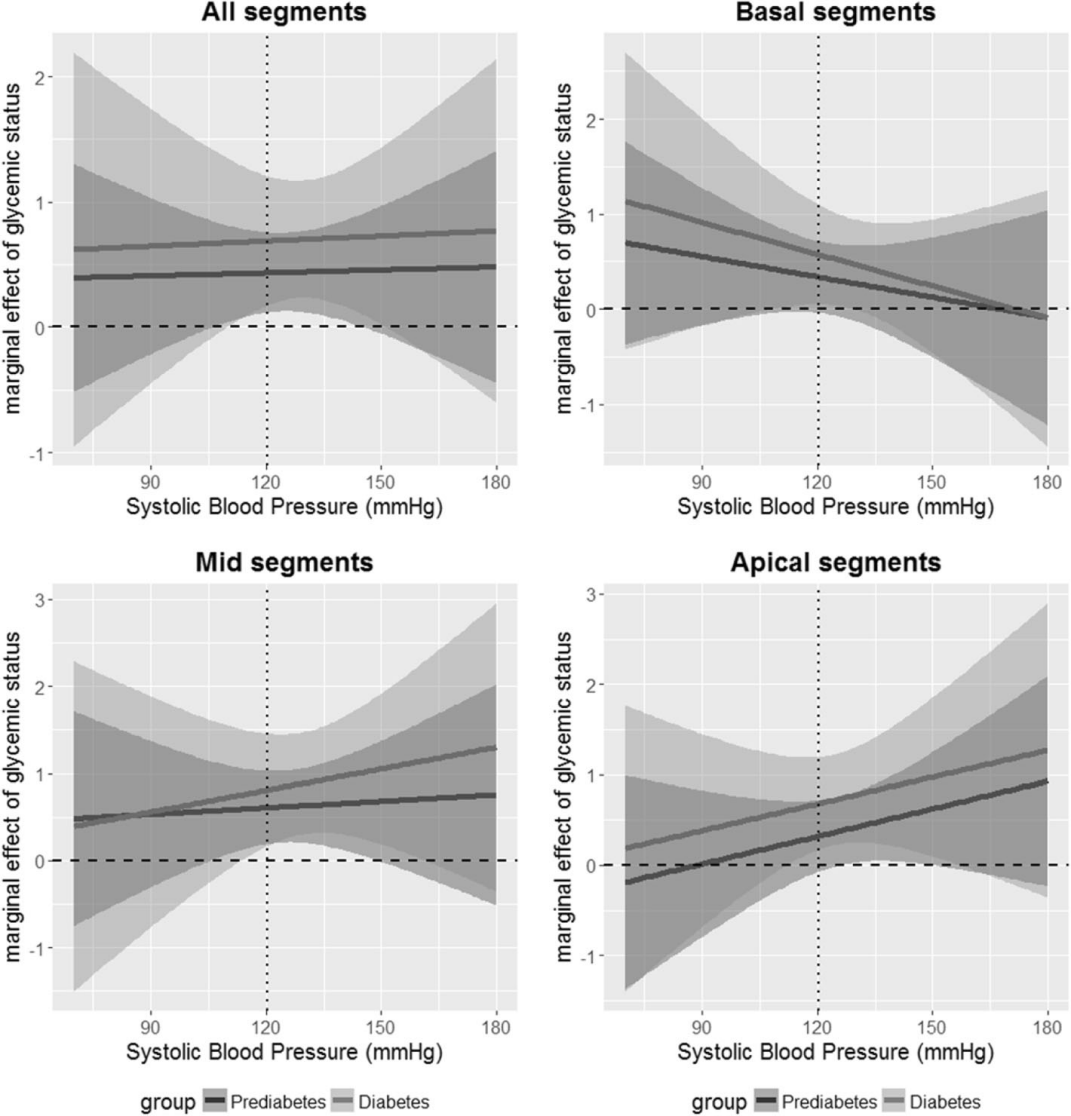
Marginal effects of glycemic status on wall thickness for multiplicative interactions with systolic blood pressure. Marginal effects indicate the size of the association of the respective group (prediabetes or diabetes) with wall thickness for a specific value of systolic blood pressure. Displayed are the marginal effects of prediabetes (solid line, dark grey) and diabetes (solid line, light grey) and the respective 95% confidence interval for a grid of possible values of systolic blood pressure (range in data: 73–179.5 mmHg). The arithmetic mean is indicated by a dotted line. The dashed line indicates the line of no effect

As displayed in Fig. 2 we found no interaction of glycemic status and systolic blood pressure for mean wall thickness averaged over all segments. Marginal effects of glycemic status, i.e. associations of prediabetes and diabetes with wall thickness for a specific value of systolic blood pressure, remained constant over the range of possible blood pressure values. However, for basal segments, there was a decreasing marginal effect of glycemic status with rising blood pressure, whereas for mid-cavity and apical segments the marginal effect of glycemic status was increasing with rising blood pressure.

## Discussion

Increased LV wall thickness is associated with a higher risk for cardiovascular outcomes. Recent findings from the Framingham study showed that a 0.1 unit increase in relative wall thickness was accompanied by a 59% increase in the hazard for cardiovascular disease [1]. A detailed assessment of LV geometry based on regional wall thickness can predict risk of incident cardiovascular disease [26]. Given these implications, it is of major importance to determine modifiable risk factors for increased wall thickness.

Our findings from this cross-sectional study show that (i) type 2 diabetes is associated to increased LV wall thickness, independent of traditional cardiovascular risk factors and especially independent of hypertension, (ii) the independent association of abnormal glucose homeostasis to cardiac structure is already present in prediabetes, (iii) individual LV segments are differently affected by hypertension and glycemic status. Thereby, we could demonstrate that the more specific evaluation of CMR derived regional LV wall thickness unveils associations that cannot be detected when assessing LV mass alone.

Our results therefore support and extend findings from other established population-based studies. In the Atherosclerosis Risk in the Community (ARIC) study, Skali et al. found that mean and relative LV wall thickness were elevated in subjects with diabetes independent of systolic blood pressure. To a certain extent, wall thickness was already increased in subjects with prediabetes [27]. In the Framingham Offspring Cohort, Velageti et al. [28] found an increasing CMR derived relative wall thickness across glycemia categories. After multivariable adjustment, the association remained significant in men. In the Multiethnic Study of Atherosclerosis (MESA), an association between CMR-derived LV mass and diabetes was found that was independent of blood pressure; however no measurements of segmental wall thickness were taken [29]. On the other hand, Bertoni et al. measured mid-cavity segments and found an increasing wall thickness for Caucasian subjects with normal glucose metabolism to subjects with iIFG and type 2 diabetes. The differences were not significant after adjustment for other cardiovascular risk factors [30]. Another study found an association of glycemic status to LV mass, but not to (relative or mean) wall thickness [31]. In our sample, we showed that the increased LV mass in subjects with diabetes was attributable to hypertension but there are independent regional associations of diabetes and blood pressure in segmental wall thickness.

Regarding the prediabetic state, recent evidence from the CARDIA study implies that longer exposure to abnormal glucose tolerance, longer duration of diabetes, and early onset of diabetes leads to more unfavorable remodeling [32]. Analyses from the Strong Heart Study demonstrated increased LV mass in Native Americans with impaired glucose tolerance, however the finding was less definitive for measures of wall thickness [33]. Our findings corroborate that prediabetes, defined as either iIGT, iIFG or IFG + IGT, is independently associated to increased wall thickness. Our sample size was probably too small to detect gradual effects of these different prediabetic groups.

Disentangling the associations of blood pressure and glycemic status proves to be complicated. In the Strong Heart Study, LV mass of subjects with diabetes but without hypertension was significantly lower compared to those subjects with both conditions, whereas relative wall thickness was not different [34]. In the HyperGEN study comprising only hypertensive subjects, diabetes was independently associated to increased LV mass [35]. A recent Chinese study suggested additive effects of diabetes and hypertension to LV remodeling [36]. Although blood pressure reduction appears to be an effective way of lowering the risk of cardiovascular disease in hypertensive patients with increased LV mass, [37] these treatments do not seem to be as effective in patients with diabetes [38]. In our study, we found a decreasing marginal effect of both prediabetes and diabetes with rising blood pressure for the basal segments, whereas the marginal effect for apical and mid segments was increasing. Thus we could further characterize the complex interplay of blood pressure and glycemic status and its impact on cardiac geometry.

The exact mechanisms of impaired glucose metabolism on LV geometry remain to be identified. Increased LV stiffness, induced by an accumulation of collagen and advanced glycation end products and subsequent fibrosis in diabetic cardiomyopathy have been suggested to contribute to LV remodeling [39, 40]. Also, a decreased myocardial perfusion reserve in subjects with diabetes induced by an impaired myocardial blood flow has been shown to be correlated with LV torsion and strain [41]. Recent findings imply that myocardial steatosis, excess storage of cardiac triglycerides, and impaired myocardial energetics are associated with concentric LV remodeling [42]. However, our study is limited in this respect as it cannot explain the pathophysiological mechanisms behind the association of glycemic status on regional LV geometry.

The values of LV wall thickness reported here, as measured by short-axis cine SSFP, substantially exceed the reference values according to the 16-segment model suggested by other groups [43, 44]. These reference values have been obtained from healthy subjects with a low-risk profile for developing cardiovascular disease, excluding smokers, and people with hypertension or diabetes. Given the major impact of these risk factors on wall thickness, it is plausible that our study found larger values for the control group.

For this nested cross-sectional study, we used a well-characterized population-based cohort. Highly standardized measurements and validation allowed us to precisely define covariates and glycemic status. Furthermore, using adequate sampling weights we were able to relate our results to the full underlying cohort.

Limitations of our study include its cross-sectional design that precludes causal inference. Further longitudinal follow-up of this study sample is mandated to determine the prognostic potential of segmental wall thickness.

## Conclusion

Our findings highlight the role of glycemic status as a potential risk factor and implicate prediabetes as unfavorably associated to LV wall thickness. Measurements of regional wall thickness provides a more precise picture than assessing overall myocardial mass. Delaying or halting progression from impaired fasting glucose to diabetes might prevent further thickening of the ventricular walls and subsequent cardiovascular complications.

## Additional file

Additional file 1: **Text S1.** Description of laboratory measurements. **Figure S1.** Intra-and interobserver agreement. **Text S2.** Description of the underlying eligible cohort. **Table S1.** Characteristics of study subjects from the full eligible cohort used for the calculation of sampling weights. **Table S2.** Associations of glycemic status with wall thickness from weighted and unweighted linear regression models. **Figure S2.** Mean wall thickness according to prediabetic glycemic status. **Table S3.** Association of prediabetic glycemic status and mean wall thickness. (DOCX 57 kb)

## Abbreviations

(i)IFG: (isolated) impaired fasting glucose
(i)IGT: (isolated) impaired glucose tolerance
AHA: American Heart Association
BMI: Body Mass Index
CI: Confidence interval
CMR: Cardiac magnetic resonance
KORA: Cooperative Health Research in the Region of Augsburg
LV: Left ventricular
MRI: Magnetic resonance imaging
OGTT: Oral glucose tolerance test
SSFP: Steady-state free precession
WHO: World Health Organization
