# Supplementary material for: Association of glycemic status and segmental left ventricular wall thickness in subjects without prior cardiovascular disease: a cross-sectional study
Source: BMC Cardiovasc Disord. 2018 Aug 9;18:162. doi: 10.1186/s12872-018-0900-7 (PMC6085649; doi:10.1186/s12872-018-0900-7)
Supplement: Supplementary file 1 — Text S1. Description of laboratory measurements. Figure S1. Intra-and interobserver agreement. Text S2. Description of the underlying eligible cohort. Table S1. Characteristics of study subjects from the full eligible cohort used for the calculation of sampling weights. Table S2. Associations of glycemic status with wall thickness from weighted and unweighted linear regression models. Figure S2. Mean wall thickness according to prediabetic glycemic status. Table S3. Association of prediabetic glycemic status and mean wall thickness. (DOCX 57 kb) [file 12872_2018_900_MOESM1_ESM.docx]

**Additional file**

**Text S1: Description of laboratory measurements**

Glucose, total cholesterol, HDL cholesterol, LDL cholesterol as well as triglyceride levels were measured in fresh serum by enzymatic, colorimetric methods using GLU, CHOL, LDLC, HDLC, and TRIG Flex assays, respectively, on a Dimension Vista 1500 instrument (Siemens Healthcare Diagnostics Inc., Newark, USA) or using GLUC3, CHOL2, LDL_C, HDLC3, and TRIGL assays, respectively, on a Cobas c702 instrument (Roche Diagnostics GmbH, Mannheim, Germany). Insulin was measured in fresh serum by an solid-phase enzyme-labeled chemiluminescent immunometric assay on an Immulite 2000 systems analyzer (Siemens) or by an electrochemiluminescence immunoassay on a Cobas e602 instrument (Roche). The measurement instrument and assays changed from Siemens to Roche halfway during the study. Calibration formulas were developed using 122 (194 for insulin) KORA FF4 samples which were measured with both methods during the time of the change. The Siemens measurement results were calibrated to the Roche measurements using the following formulas [insulin in µU/mL; all other units in mg/dL]: Total_Cholesterol_Roche = 3.00 + Total_Cholesterol_Siemens * 1.00; HDL_Cholesterol_Roche = 2.40 + HDL_Cholesterol_Siemens * 1.12; LDL_Cholesterol_Roche = antilog (-0.13328 + log LDL_Cholesterol_Siemens * 1.03051); Triglycerides_Roche = 4.97073 + Triglycerides_Siemens * 0.90732; Insulin_Roche = 1.307 + Insulin_Siemens * 1.016. No calibration was needed for glucose because the double measurements were very similar so that the intercept and the slope of the Passing-Bablok regression used for calibration were estimated to be zero and one, respectively. HbA1c was measured in hemolyzed whole blood using the cation-exchange high performance liquid chromatographic, photometric VARIANT II TURBO HbA1c Kit - 2.0 assay on a VARIANT II TURBO Hemoglobin Testing System (Bio-Rad Laboratories Inc., Hercules, USA).

**Figure S1: Intra-and interobserver agreement**


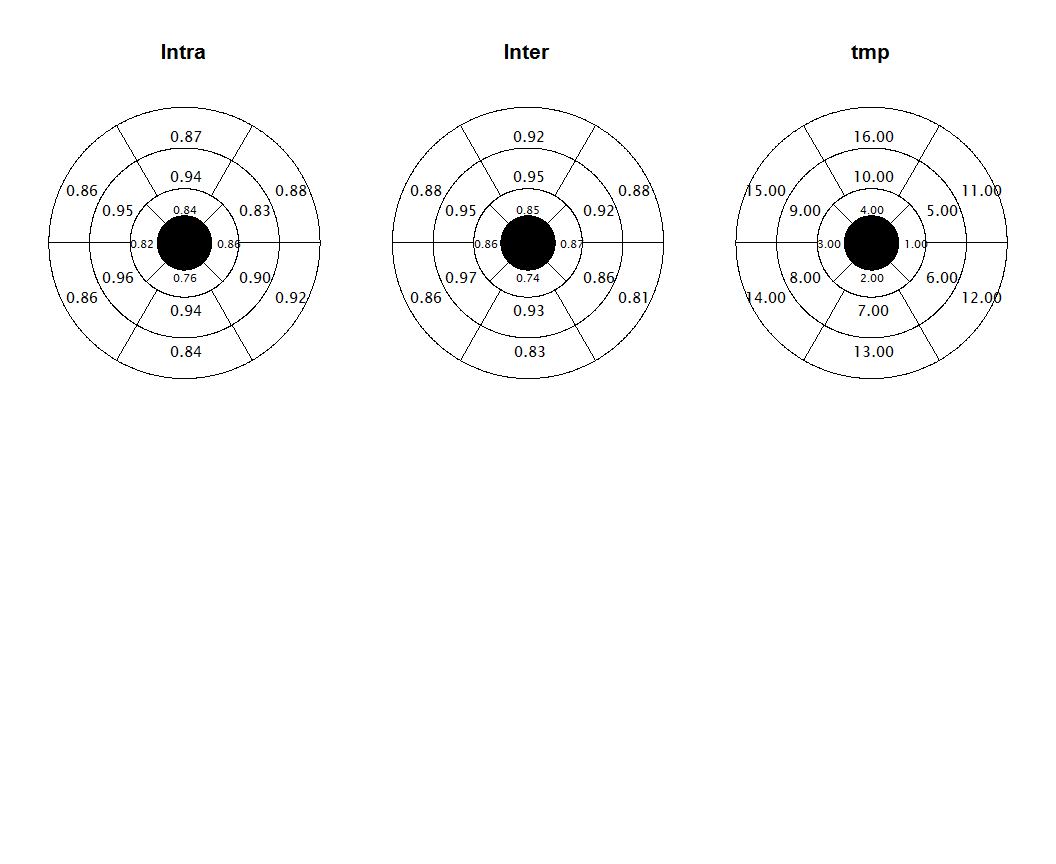


Intraclass Correlation Coefficients for intra- and interobserver agreement for end-diastolic wall thickness. Intraobserver agreement was based on 25 subjects and interobserver agreement based on 52 subjects.

**Text S2: Description of the underlying eligible cohort**

The original KORA FF4 cohort comprises N = 2279 participants, whereas the MRI study sample comprises N = 400 participants. Due to the sampling design of the MRI study, not all subjects from the cohort can be represented by a subject from the MRI sample. The underlying eligible cohort comprises n = 1652 subjects: not eligible were subjects older than 72 years (n = 428), subjects with indeterminable glycemic status (n = 69), a history of myocardial infarction or stroke (n = 63), subjects with implanted medical devices (n = 11) and subjects with impaired renal function (n = 56). We treated the remaining n = 1652 subjects as the underlying cohort for the calculation of sampling weights. The difference to the n = 1282 subjects as presented in (1) arises because we considered the exclusion criteria claustrophobia, pregnancy, inability to hold breath, tattoos and allergy to contrast agents as unrelated to a subject’s glycemic status and cardiac morphology. Therefore, all subjects for whom these exclusion criteria would apply can nevertheless be represented in their glycemic status and cardiac morphology by subjects who participated in the MRI study. Base weights were calculated by inverse-probability cell weighting in 12 cells defined by glycemic status and age category (38-47 years, 48 – 57 years, 58 – 67 years, 68- 72 years) (2) and were then further modified by post-stratification according to sex (3). Weighted variances and standard errors were computed by Taylor series linearization.

**Table S1: Characteristics of study subjects from the full eligible cohort used for the calculation of sampling weights.**

|  | all | Control | Prediabetes | Type 2 Diabetes |
| --- | --- | --- | --- | --- |
|  | N = 1652 | N = 1240 | N = 250 | N = 162 |
| Age (years) | 56.1 ± 9.4 | 54.3 ± 9.0 | 60.5 ± 8.9 | 62.7 ± 8.4 |
| Male gender n (%) | 763 (46.2%) | 538 (43.4%) | 136 (54.4%) | 89 (54.9%) |
| BMI (kg/m^2^) | 27.6 ± 5.1 | 26.5 ± 4.5 | 30.2 ± 5.2 | 31.5 ± 5.6 |
| Systolic BP (mmHg) | 118.0 ± 16.9 | 115.5 ± 15.8 | 123.9 ± 16.4 | 127.6 ± 19.7 |
| Hypertension n (%) | 497 (30.1%) | 272 (21.9%) | 116 (46.4%) | 109 (67.3%) |
| Total Cholesterol (mg/dL) | 219.2 ± 37.6 | 219.2 ± 37.0 | 224.4 ± 37.5 | 211.0 ± 40.4 |
| Smoking n (%) | |  |  |  |
| never-smoker | 726 (43.9%) | 555 (44.8%) | 98 (39.2%) | 73 (45.1%) |
| ex-smoker | 625 (37.8%) | 449 (36.2%) | 107 (42.8%) | 69 (42.6%) |
| smoker | 301 (18.2%) | 236 (19.0%) | 45 (18%) | 20 (12.3%) |

**Table S2:** Associations of glycemic status with wall thickness from weighted and unweighted linear regression models.

|  |  | **Prediabetes** | | | **Diabetes** | | |
| --- | --- | --- | --- | --- | --- | --- | --- |
|  |  | estimate | 95%-CI | p-value | estimate | 95%-CI | p-value |
| **Diastole** |  |  |  |  |  |  |  |
| Wall thickness (mm): arithmetic mean of | | |  |  |  |  |  |
| all segments | unweighted | 0.36 | [0.07, 0.65] | **0.014** | 0.69 | [0.31, 1.08] | **<0.001** |
|  | weighted | 0.44 | [0.12, 0.75] | **0.007** | 0.70 | [0.23, 1.17] | **0.004** |
| basal segments | unweighted | 0.32 | [-0.02, 0.66] | 0.065 | 0.53 | [0.08, 0.98] | **0.022** |
|  | weighted | 0.33 | [-0.05, 0.70] | 0.087 | 0.51 | [0.02, 0.99] | **0.040** |
| mid segments | unweighted | 0.49 | [0.13, 0.85] | **0.007** | 0.86 | [0.39, 1.34] | **<0.001** |
|  | weighted | 0.61 | [0.20, 1.02] | **0.004** | 0.86 | [0.28, 1.45] | **0.004** |
| apical segments | unweighted | 0.24 | [-0.11, 0.60] | 0.178 | 0.69 | [0.22, 1.17] | **0.004** |
|  | weighted | 0.34 | [-0.04, 0.73] | 0.080 | 0.74 | [0.23, 1.24] | **0.005** |
| lateral segments | unweighted | 0.38 | [0.05, 0.71] | **0.024** | 0.64 | [0.20, 1.08] | **0.005** |
|  | weighted | 0.46 | [0.09, 0.83] | **0.014** | 0.65 | [0.14, 1.16] | **0.013** |
| septal segments | unweighted | 0.31 | [0.02, 0.61] | **0.035** | 0.66 | [0.27, 1.05] | **0.001** |
|  | weighted | 0.35 | [0.05, 0.65] | **0.023** | 0.64 | [0.18, 1.10] | **0.006** |
| anterior segments | unweighted | 0.44 | [0.05, 0.83] | **0.027** | 1.00 | [0.48, 1.52] | **<0.001** |
|  | weighted | 0.52 | [0.10, 0.93] | **0.015** | 0.98 | [0.38, 1.59] | **0.002** |
| inferior segments | unweighted | 0.35 | [0.04, 0.66] | **0.028** | 0.54 | [0.13, 0.96] | **0.011** |
|  | weighted | 0.45 | [0.11, 0.79] | **0.009** | 0.58 | [0.11, 1.04] | **0.016** |
| myocardial mass (g/m^2^) | unweighted | -1.37 | [-4.52, 1.77] | 0.392 | 0.00 | [-4.20, 4.20] | 1 |
|  | weighted | -0.11 | [-3.51, 3.28] | 0.948 | 0.56 | [-4.94, 6.07] | 0.841 |

**Figure S2:** Mean wall thickness according to prediabetic glycemic status.

iIFG: isolated impaired fasting glucose. iIGT: isolated impaired glucose tolerance. IFG+IGT: both impaired fasting glucose and impaired glucose tolerance.


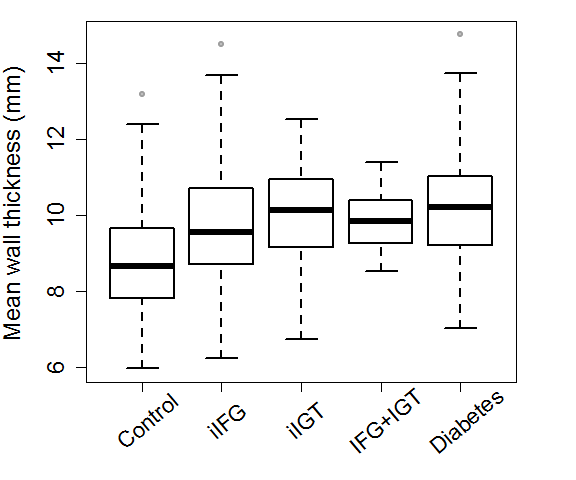


**Table S3:** Association of prediabetic glycemic status on mean wall thickness.

|  | **isolated IFG (N = 35)** | | | **isolated IGT (N = 41)** | | | **IFG + IGT (N = 16)** | | |
| --- | --- | --- | --- | --- | --- | --- | --- | --- | --- |
|  | estimate | 95%-CI | p-value | estimate | 95%-CI | p-value | estimate | 95%-CI | p-value |
| Wall thickness (mm): arithmetic mean of |  |  |  |  |  |  |  |  |  |
| all segments | 0.50 | [-0.03, 1.04] | 0.067 | 0.39 | [0.02, 0.76] | 0.038 | 0.41 | [0.01, 0.81] | 0.047 |
| basal segments | 0.28 | [-0.23, 0.80] | 0.278 | 0.17 | [-0.31, 0.64] | 0.490 | 0.77 | [0.04, 1.50] | 0.118 |
| mid segments | 0.75 | [0.09, 1.41] | 0.082 | 0.61 | [0.05, 1.17] | 0.098 | 0.33 | [-0.20, 0.86] | 0.326 |
| apical segments | 0.46 | [-0.24, 1.15] | 0.278 | 0.41 | [-0.03, 0.84] | 0.099 | -0.03 | [-0.51, 0.46] | 0.911 |
| lateral segments | 0.45 | [-0.23, 1.12] | 0.193 | 0.48 | [0.04, 0.91] | 0.133 | 0.46 | [-0.04, 0.97] | 0.149 |
| septal segments | 0.44 | [-0.01, 0.90] | 0.092 | 0.29 | [-0.10, 0.68] | 0.141 | 0.31 | [-0.17, 0.79] | 0.207 |
| anterior segments | 0.68 | [-0.05, 1.41] | 0.092 | 0.46 | [-0.03, 0.94] | 0.133 | 0.33 | [-0.17, 0.83] | 0.207 |
| inferior segments | 0.51 | [0.02, 0.99] | 0.092 | 0.36 | [-0.08, 0.80] | 0.141 | 0.56 | [0.10, 1.03] | 0.074 |
| myocardial mass (g/m^2^) | 0.88 | [-4.81, 6.57] | 0.762 | -0.89 | [-4.92, 3.14] | 0.665 | -0.27 | [-5.69, 5.15] | 0.923 |

Estimates from linear regression models adjusted for age, sex, BMI, systolic BP, total cholesterol, use of antihypertensive medication and smoking status.CI: Confidence Interval. Results of the diabetes group are not displayed as the interpretation remains unchanged compared to Table 3 in the main manuscript

1. Bamberg F, Hetterich H, Rospleszcz S, Lorbeer R, Auweter SD, Schlett CL, et al. Subclinical Disease in Subjects with Prediabetes, Diabetes and Normal Controls from the General Population: the KORA MRI-Study. Diabetes. 2016.

2. Kalton G, Flores-Cervantes I. Weighting methods. Journal of Official Statistics. 2003;19(2):81.

3. Little RJ. Post-stratification: a modeler's perspective. Journal of the American Statistical Association. 1993;88(423):1001-12.
